# Reply to “Utility of Unrefined Carbohydrates in Type 2 Diabetes. Comment on Reversing Type 2 Diabetes: A Narrative Review of the Evidence, *Nutrients*, 2019, *11*, 766”

**DOI:** 10.3390/nu11071644

**Published:** 2019-07-18

**Authors:** Sarah J Hallberg, Victoria M Gershuni, Tamara L Hazbun, Shaminie J Athinarayanan

**Affiliations:** 1 Virta Health, 535 Mission Street, San Francisco, CA 94105, USA; shaminie@virtahealth.com; 2 Indiana University Health Arnett, Lafayette, IN 47904, USA; thazbun@iuhealth.org; 3 Indiana University School of Medicine, Indianapolis, IN 46202, USA; 4 Department of Surgery, Perelman School of Medicine University of Pennsylvania, Philadelphia, PA 19104, USA; victoriagershunimd@gmail.com

We appreciate the interest and comments from Joshi et al. [1] regarding our recent paper [2]. Here are our specific comments to concerns that they raised.

First, the authors cite a paper by Anderson et al. [3] that demonstrated diabetes reversal (unclear if all patients had type 2 diabetes) with a higher carbohydrate diet; however, a significant limitation of the Anderson paper was that the available data shows a decline of fasting glucose even while on the control diet, which was implemented first. This meaningful improvement in glycemia may signify a change from their baseline diet—specifically sucrose intake, which was eliminated in both the control and higher carbohydrate intervention diets. Furthermore, the high-carbohydrate intervention in the study by Anderson et al. was performed in a fully controlled metabolic ward setting, where caloric intake was controlled, and the diets were administered by hospital staff. One of the limitations of the paper is that they did not discuss whether there were differences in caloric intake in the patients’ diet (in both control and high-carbohydrate diets) compared to what they usually consume on a daily basis. While significant change in weight was not observed in the study participants, the diet was administered for a very short period of time, and the participants started off with a lean baseline weight, which may account for the non-significant weight change in this group. Together, the improvements in glycemia observed in patients while on a control diet, the short duration of the study, and the artificial constructs of a metabolic ward study makes it difficult to draw a clear conclusion regarding the impact of an ad libitum high-carbohydrate diet intervention. 

Second, Joshi et al. [1] point to a study by Rosenbaum and colleagues that demonstrated higher post-prandial glucose levels; however, Joshi et al. failed to mention that these values were measured following a moderate carbohydrate meal in the context of consuming a ketogenic diet [4]. It has been previously documented that in as little as three days, a low-carbohydrate, high-fat (LCHF) diet will challenge the first phase insulin response to a higher-carb meal, resulting in a greater initial insulin peak, and therefore, these results are not surprising [5]. Also, not surprising (nor mentioned) is the finding of blunted glucose levels and insulin response to the ketogenic meal among participants fed both the baseline and the ketogenic diets, which demonstrates that a ketogenic diet may be beneficial in all patients for avoiding post-prandial glucose excursions. Furthermore, this study did not involve persons with type 2 diabetes, so it was not appropriate to mention in our review of the literature that specifically involved type 2 diabetes. 

The third comment involved concerns the differing levels of support in the Saslow et al. [6] and Hallberg et al. [7] trials, which was acknowledged in both of the papers (including the title of the Saslow paper). Both studies were designed to examine an intervention compared to the current standard of care. Differing levels of support is commonplace in lifestyle intervention trials such as LOOK AHEAD [8]. 

Fourth, the concerns raised about the findings from Hallberg et al. [7] are riddled with factual errors. Hallberg et al. [7] found a DM reversal rate in response to a ketogenic diet of 60%; reversal was defined as HbA1c below 6.5% and discontinuation of all diabetes-specific medications (excluding metformin), not 78% as stated in the Joshi comments. Due to a baseline diagnosis of type 2 diabetes, all patients in the Hallberg study, even after reversal of DM, are at high risk for developing diabetes in the future. Thus, metformin was continued in these patients as an adjunct that is well-known to decrease the risk for developing diabetes [9]. The inclusion of metformin may preclude the currently accepted definition for remission but does not diminish the reversal of DM that accompanied a ketogenic diet along with this choice being appropriate individual patient care. For clarification, in the Hallberg study, the starting HbA1c was 7.6%, not 6.6%, and the net improvement at one year of the intervention over the control was 1.3%, not 0.3%, both of which were erroneously reported in the Joshi comments. Lastly, all conflicts of interest were fully declared for this paper in accordance to guidelines. 

Furthermore, this review was not intended to be a systematic review, and therefore does not reflect the entirety of the current evidence. However, we feel that this review gives an accurate depiction of the evidence that exists for diabetes reversal. Joshi et al. present no evidence to support the title of their paper (presumably their point) *Utility of Unrefined Carbohydrates in Type 2 Diabetes. Comment on Reversing Type 2 Diabetes: A Narrative Review of the Evidence*, *Nutrients* 2019, *11*, 766 [1].

We hope the above gives clarity to the issues raised by Joshi et al. [1]. We appreciated the opportunity to reply to questions about our work.
